# Effectiveness of Antiviral Treatment with Intravenous Peramivir and Oral Oseltamivir for Seasonal Influenza in Children

**DOI:** 10.3390/children12010026

**Published:** 2024-12-27

**Authors:** Young-hoon Byun, Ji-Eun Kim, So-Hyun Paek, Min-Jung Kim, Soo Hyun Park, Ho-Young Song, Jaehyun Kwon

**Affiliations:** 1 Department of Emergency Medicine, CHA Bundang Medical Center, School of Medicine, CHA University, Seongnam 13496, Republic of Korea; byunyoun84@chamc.co.kr (Y.-h.B.); hyun21400@naver.com (S.-H.P.); mjtear@naver.com (M.-J.K.); suas11@cha.ac.kr (S.H.P.); shyped85@chamc.co.kr (H.-Y.S.); 2 Department of Emergency Medicine, Dong-A University Hospital, Busan 49201, Republic of Korea; amcfsapple@dau.ac.kr

**Keywords:** influenza, peramivir, pediatric emergency department

## Abstract

Background/Objectives: Influenza poses significant risks in children, causing complications like febrile seizures and hospitalizations. Antiviral treatments include oseltamivir and peramivir, both FDA-approved neuraminidase inhibitors. This study aims to compare the effectiveness of intravenous peramivir and oral oseltamivir in pediatric patients presenting to an emergency department, with a primary focus on the revisit rate within 72 h post-treatment. Methods: A retrospective study analyzed 1327 children aged 1–15 years diagnosed with influenza A or B between 1 January 2019 and 29 February 2020, at a single urban hospital. Patients were divided into oseltamivir (n = 1243) and peramivir (n = 84) groups. Data included demographics, clinical symptoms, emergency department stays, and revisit rates. Fisher’s exact test was used for analysis, with *p*-values < 0.05 considered significant. Results: A total of 1327 pediatric patients were included, with 1243 receiving oseltamivir and 84 receiving peramivir. Patients in the peramivir group were older (median age 5.88 years vs. 4.54 years, *p* = 0.002) and had higher rates of gastrointestinal symptoms. The emergency department length of stay was significantly longer in the peramivir group (167 min vs. 63 min, *p* < 0.001). The revisit rate within 3 days was 5.63% for oseltamivir and 9.52% for peramivir, with no statistically significant difference (*p* = 0.22). Conclusions: Peramivir presents as an effective alternative treatment for influenza in children, particularly in situations where oral administration is not feasible due to gastrointestinal intolerance, highlighting the importance of an alternative route of antiviral administration.

## 1. Introduction

Seasonal influenza is a common respiratory pathogen that affects 20–30% of children worldwide annually [1]. Due to its widespread prevalence in autumn and winter, influenza performs a significant role in public health [2,3]. Influenza accounts for approximately 10% of respiratory hospitalizations among children under 18 years of age, with rates varying by age group: 5% in infants under 6 months and 16% in children aged 5–17 years [2].

Symptoms of influenza range from mild respiratory symptoms to systemic symptoms such as fever, headache, and myalgia, depending on the characteristics of the host and the virus. Among the four types of influenza viruses (A, B, C, D), type A and B are the main types causing seasonal epidemics [1]. Influenza A and B generally present as acute, self-limited illnesses in healthy individuals; however, in some cases, they may cause complications such as lower respiratory tract infections and encephalopathy. Children, in particular, may experience higher peak temperatures, febrile seizures, and myositis compared to adults, and children under 5 are at increased risk of hospitalization [4,5,6,7].

For these reasons, influenza vaccination is strongly recommended for children, but there remains a tendency for some children not to vaccinate against influenza [8,9]. Therefore, antiviral treatment is crucial for managing influenza infection in the vulnerable pediatric population [10]. The CDC recommends antiviral treatment for individuals infected with influenza to reduce severe complications and shorten the course of illness, especially for high-risk groups, such as children, adults over 65, pregnant women, and those with specific comorbidities [11,12]. The most common antiviral agents used in the treatment of influenza in pediatric emergency settings are neuraminidase inhibitors, such as oseltamivir and peramivir, both of which have been approved by the U.S. Food and Drug Administration (FDA) for the treatment of acute, uncomplicated influenza in children aged two weeks and older (oseltamivir) and six months and older (peramivir) [13,14,15,16,17]. Oseltamivir is administered orally and converted into an active metabolite in the liver, requiring continuous dosing. In contrast, peramivir is administered intravenously in its active form, providing rapid action with a single dose, which is particularly useful in acute settings. These differences influence the route of administration and clinical preference for each drug [4].

Despite the frequent use of these antivirals in emergency departments, research comparing their effectiveness in reducing the need for repeated emergency department visits, particularly in the pediatric population, is scarce. Most studies evaluating the clinical efficacy of oseltamivir and peramivir in children are based on small cohorts and focus mainly on inpatients or outpatients [10,18,19,20,21,22,23,24,25]. This study aims to compare the therapeutic effects of intravenous peramivir and oral oseltamivir in pediatric patients who visited a pediatric emergency department. The main outcome of this retrospective study is the emergency department revisit rate within 72 h after treatment with peramivir or oseltamivir. Understanding the effectiveness of these antiviral treatments in preventing revisits is imperative to optimize influenza treatment strategies for children in emergency settings.

## 2. Materials and Methods

### 2.1. Data Source and Setting

This study targeted pediatric patients aged between 1 and 15 years who visited a pediatric emergency department at a single urban resident-training hospital and were diagnosed with influenza type A or B. Researchers reviewed electronic medical records (EMR) to collect and analyze medical information of patients who visited this hospital during a 14-month period from 1 January 2019 to 29 February 2020.

### 2.2. Data Collection

We collected patients’ demographic information (gender, age) as well as vital signs (blood pressure, pulse rate, respiratory rate, oxygen saturation, and body temperature) and assessed respiratory, gastrointestinal, and neurological symptoms reported by patients. For fever, we recorded the time elapsed from the onset of body temperature above 38 °C until the emergency department visit, categorizing the time as follows: 0–24 h as 1 day, 24–48 h as 2 days, and 48–72 h as 3 days. Respiratory symptoms included cough, sputum, and rhinorrhea; gastrointestinal symptoms included nausea, vomiting, diarrhea, and abdominal pain; neurological symptoms included headache, dizziness, seizure, and hallucination. Diagnosis of influenza was confirmed using a rapid antigen test (nasopharyngeal secretions were collected and tested using COPAN FLOQSwabs (HGT044.310 Rev.00)), and influenza type was classified as type A or B based on ICD-10 codes. Patients who received peramivir and those who received oseltamivir were categorized into respective treatment groups. In each group, we investigated revisit rates within 72 h and emergency department length of stay (EDLOS). Revisits not related to the initial visit were not considered as revisits. Clinical outcomes were categorized as discharge, transfer, hospitalization, death, or others (e.g., voluntary discharge), and all outcomes were based on the results from the first visit, excluding the results from revisits. Laboratory findings included blood test results, and radiologic findings referred to chest X-ray findings. We also investigated the complication rates of patients who revisited within 3 days, including persistent fever, gastrointestinal symptoms, respiratory symptoms, and neurological symptoms. For peramivir, Peramiflu Premix Inj. (peramivir hydrate) was administered at a dose of 10 mg/kg as a single dose via intravenous infusion over at least 15 min, with a maximum dose of 600 mg. For oseltamivir, Tamiflu Cap. (oseltamivir phosphate) was administered orally at a dose of 2 mg/kg twice daily for 5 days (a total of 10 doses), with a maximum dose of 75 mg.

### 2.3. Statistical Analysis

For data processing and statistical analyses, we employed R version 4.2.1 (R Foundation for Statistical Computing, Vienna, Austria, 2022). Categorical variables are presented in terms of frequency distributions (%), whereas continuous variables are characterized using medians and interquartile ranges (IQR). *p*-values derived from Fisher’s exact test that were less than 0.05 were deemed statistically significant.

### 2.4. Ethics Statement

This study was approved by the Institutional Review Board (IRB) of Bundang CHA Hospital. Given the retrospective cross-sectional nature of the study, the requirement for obtaining consent from study participants was waived.

## 3. Results

**Figure 1:**
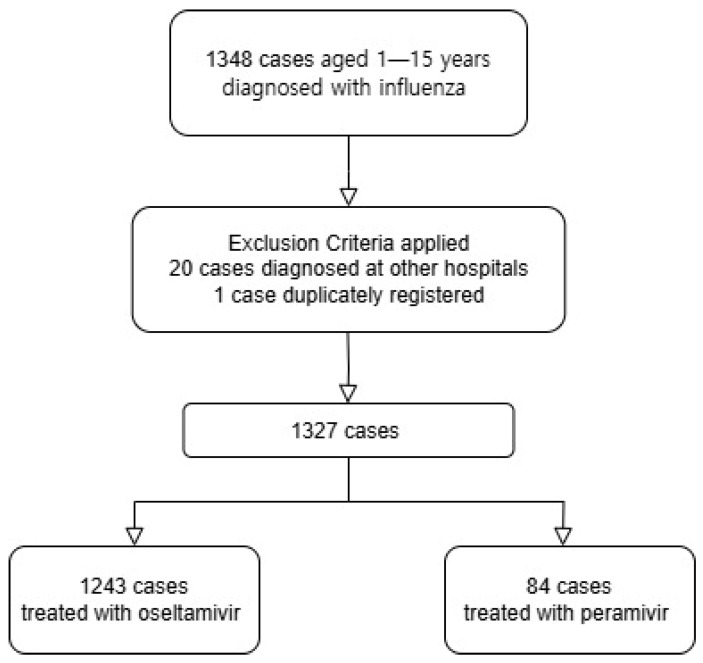
Flow chart of patients’ characteristics.

Among the patients aged between 1 and 15 years who visited this hospital from 1 January 2019 to 29 February 2020, a total of 1348 patients were diagnosed with influenza. Upon reviewing the medical records, 20 patients were diagnosed with influenza at facilities other than this hospital, and 1 patient had a duplicate entry, resulting in a total of 1327 patients included in this study. The characteristics of the included patients are summarized in Figure 1.

The characteristics of the patients included in the oseltamivir and peramivir groups are presented in Table 1. Out of the total 1327 patients, 1243 (93.67%) were in the oseltamivir group, and 84 (6.33%) were in the peramivir group. The median age of patients in the oseltamivir group was 4.54 years (IQR: 2.75–6.75, mean: 5.04 ± 3.18), while the median age in the peramivir group was 5.88 years (IQR: 4.00–7.56, mean: 6.17 ± 3.15), presenting a statistically significant difference between the two groups (*p* = 0.002). The proportion of male patients was similar between the two groups, with 51.65% in the oseltamivir group and 53.57% in the peramivir group (*p* = 0.91).

With respect to influenza type, influenza A was more common in both groups, accounting for 71.36% in the oseltamivir group and 73.81% in the peramivir group, with no significant difference (*p* = 0.71). The frequency of blood tests and chest X-rays differed significantly between the two groups. Chest X-rays were performed in 48.11% of patients in the oseltamivir group and 67.86% in the peramivir group (*p* < 0.001). Blood tests were conducted in 11.75% of the oseltamivir group and 84.52% of the peramivir group (*p* < 0.001).

The median duration of fever was 1 day for both groups (mean duration: 1.46 ± 0.78 days in the oseltamivir group, 1.54 ± 0.87 days in the peramivir group), with no significant difference (*p* = 0.41). Regarding clinical symptoms, gastrointestinal symptoms were more common in the peramivir group (58.33%) compared to those in the oseltamivir group (27.35%) (*p* < 0.001). Respiratory symptoms were observed in 87.93% of the oseltamivir group and 80.95% of the peramivir group, with no significant difference (*p* = 0.15). Neurological symptoms were reported in 12.79% of the oseltamivir group and in 16.67% of the peramivir group, with no significant difference (*p* = 0.15).

The median emergency department length of stay (EDLOS) was significantly longer in the peramivir group at 167 min (IQR: 117.75–248.75, mean: 167 min) compared to 63 min (IQR: 49.00–89.00, mean: 63 min) in the oseltamivir group (*p* < 0.001).

In terms of patient outcomes, 98.47% of patients in the oseltamivir group were discharged, compared to 86.90% in the peramivir group. The hospitalization rate was significantly higher in the peramivir group (11.90%) compared to in the oseltamivir group (1.13%) (*p* < 0.001).

The revisit rate within 3 days was 5.63% in the oseltamivir group and 9.52% in the peramivir group, with no statistically significant difference (*p* = 0.22).

The complication rates among patients who revisited after using each antiviral drug are summarized in Table 2. Symptoms observed after drug administration were compared between the oseltamivir group (70 patients) and the peramivir group (8 patients). Persistent fever occurred in 32.9% (23 patients) of the oseltamivir group and 12.5% (1 patient) of the peramivir group, with no statistically significant difference (*p* = 0.423). Gastrointestinal symptoms were reported in 42.9% (30 patients) of the oseltamivir group and 62.5% (5 patients) of the peramivir group, with no significant difference (*p* = 0.456). Respiratory symptoms were observed in 10.0% (seven patients) of the oseltamivir group, while none were observed in the peramivir group (*p* = 1.000). Seizures occurred in 5.7% (four patients) of the oseltamivir group and 25.0% (two patients) of the peramivir group, but the difference was not statistically significant (*p* = 0.113). Skin rashes were reported in 2.9% (two patients) of the oseltamivir group, but none were observed in the peramivir group (*p* = 1.000). Psychotic symptoms were reported in 1.4% (one patient) of the oseltamivir group, while none were reported in the peramivir group (*p* = 1.000).

## 4. Discussion

This study compared the therapeutic effects of intravenous peramivir and oral oseltamivir in pediatric influenza patients visiting an emergency center. Since its approval by the FDA in 1999, oral oseltamivir has been widely used [10]; however, the requirement for twice-daily dosing over 5 days can pose challenges in acute care settings such as emergency departments, particularly for young infants or patients requiring mechanical ventilation [19,26]. In contrast, peramivir, an intravenous neuraminidase inhibitor approved by the FDA in 2013, is often used when oral administration is not feasible [10]. The convenience of single-dose intravenous administration makes peramivir an attractive alternative, particularly in emergency settings [26].

In this study, we aimed to investigate the reasons clinicians choose specific antiviral agents between peramivir and oseltamivir. The results demonstrated that the peramivir group, which requires securing an intravenous pathway, had a higher mean age and a higher rate of laboratory and radiologic tests compared to the oseltamivir group. This finding contrasts with studies conducted in pediatric intensive care units or national database studies of pediatric influenza patients, which reported no significant age differences between treatment groups [27,28].

The significant difference in the frequency of chest X-rays and blood tests performed between the two groups may also be attributable to the older age of the peramivir group, which may have facilitated additional testing. Furthermore, gastrointestinal symptoms were more frequently reported in the peramivir group, likely due to the difficulty in administering oral medication in patients with such symptoms, leading to a preference for intravenous peramivir. The EDLOS was also longer in the peramivir group, likely due to the additional X-rays and blood tests performed. Regarding discharge from the emergency department, the peramivir group had a higher rate of hospitalization compared to the oseltamivir group, which may be attributed to the common occurrence of decreased dietary intake due to gastrointestinal symptoms.

These findings are consistent with existing studies. Oseltamivir is the most frequently used oral antiviral agent for the treatment and prevention of influenza, especially since the 2009 H1N1 pandemic [26]. However, due to the challenges associated with oral administration, intravenous peramivir may be preferred as an alternative antiviral in certain cases [19]. Peramivir, with its single-dose intravenous administration, offers convenience in acute care settings and serves as an important alternative when oseltamivir cannot be administered orally [26].

We also investigated the complications among patients who revisited within 3 days after the use of either antiviral drug. The complications that occurred more frequently in the oseltamivir group compared to the peramivir group were persistent fever (32.9% vs. 12.5%), respiratory symptoms (10.0% vs. 0%), skin symptoms (2.9% vs. 0%), and hallucinations (1.4% vs. 0%). Gastrointestinal symptoms were more common in the peramivir group (42.9% vs. 62.5%). But these differences in complication rates were not statistically significant. Further research is needed to clarify whether the difference in gastrointestinal symptom rates is due to drug side effects or patient characteristics.

This study focused on the period prior to the COVID-19 pandemic to minimize selection bias. However, further research to examine the effects of the COVID-19 pandemic on the treatment of influenza may be warranted.

The main limitation of this study is that it is a single-center study, which may have introduced selection bias. Additionally, the preference of the patient’s parents regarding the choice of antiviral agent might have influenced the outcomes, potentially introducing selection bias. Moreover, the number of patients in the peramivir group was relatively smaller compared to the oseltamivir group. This discrepancy is likely because oseltamivir was more commonly employed as the first-line antiviral agent for pediatric influenza patients in South Korea during the study period. Lastly, as this study was a retrospective study, it was not possible to precisely ascertain the time required for fever to subside or to accurately assess complications caused by the antiviral agents. Furthermore, complications related to the antiviral agents in patients who did not revisit the emergency department could not be assessed, leading to loss of follow-up. However, there are few comparative studies between oseltamivir and peramivir in healthy pediatric populations, and the lack of statistically significant differences in revisit rates or adverse effects between the oseltamivir and peramivir groups adds value to this study [10,19,27]. Therefore, further studies should include larger sample sizes and employ a prospective design to thoroughly evaluate the effectiveness of peramivir and oseltamivir, particularly in severe patients, hospitalized patients, and patients presenting to the emergency department, to establish optimal antiviral treatment strategies. Additionally, further research should stratify patients into age groups (e.g., 1–5, 6–10, and 11–15 years old) to identify any differentiated characteristics and compare clinical features, such as the time required for fever to subside.

## 5. Conclusions

Intravenous peramivir demonstrates its potential as an effective alternative to oral oseltamivir for pediatric influenza treatment, particularly in cases where gastrointestinal symptoms preclude oral administration.

## Figures and Tables

**Table 1:** Comparison of baseline characteristics between the oseltamivir group and the peramivir group.

	Oseltamivir Group (N = 1243)	Peramivir Group (N = 84)	*p*-Value
Age, years			
Median, IQR	4.54 [2.75–6.75]	5.88 [4.00–7.56]	
Mean, SD	5.04 ± 3.18	6.17 ± 3.15	0.002
Gender, male	642 (51.65)	45 (53.57)	0.91
Influenza subtype			
A	887 (71.36)	62 (73.81)	
B	356 (28.64)	22 (26.19)	0.71
Performed examination			
Chest X-ray	598 (48.11)	57 (67.86)	<0.001
Laboratory test	146 (11.75)	71 (84.52)	<0.001
Fever duration, days			
Median, IQR	1 [1–2]	1 [1–2]	
Mean, SD	1.46 ± 0.78	1.54 ± 0.87	0.41
Symptoms			
Gastrointestinal	340 (27.35)	49 (58.33)	<0.001
(nausea, vomiting, diarrhea, abdominal pain)			
Respiratory			
(cough, sputum, rhinorrhea)	1093 (87.93)	68 (80.95)	0.15
Neurologic			
(headache, dizziness, seizure)	159 (12.79)	14 (16.67)	0.15
EDLOS, minutes			
Median, IQR	63 [49.00–89.00]	167 [117.75–248.75]	<0.001
Result			
Discharge	1224 (98.47)	73 (86.90)	
Hospitalization	14 (1.13)	10 (11.90)	
Others	5 (0.40)	1 (1.19)	<0.001
Revisit within 3 days	70 (5.63)	8 (9.52)	0.22

**Table 2:** Incidence of complications among revisit patients treated with oseltamivir vs. peramivir.

	Oseltamivir Group (N = 70)	Peramivir Group (N = 8)	*p*-Value
Persistent fever	23 (32.9)	1 (12.5)	0.423
GI symptoms	30 (42.9)	5 (62.5)	0.456
Respiratory symptoms	7 (10.0)	0 (0.0)	1.000
Seizure	4 (5.7)	2 (25.0)	0.113
Skin rash	2 (2.9)	0 (0.0)	1.000
Psychological symptoms	1 (1.4)	0 (0.0)	1.000

## Data Availability

The original contributions presented in this study are included in the article. Further inquiries can be directed to the corresponding author.
